# Agromorphological and Physiological Performance of Ethiopian Common Bean (*Phaseolus vulgaris* L.) Genotypes under Different Agroecological Conditions

**DOI:** 10.3390/plants12122342

**Published:** 2023-06-16

**Authors:** Shiferaw Girsil Tigist, Julia Sibiya, Assefa Amelework, Gemechu Keneni

**Affiliations:** 1 Ethiopian Institute of Agricultural Research, Melkassa Agricultural Research Centre, Adama P.O. Box 430, Ethiopia; tshiferaw2006@gmail.com; 2 African Center for Crop Improvement, University of KwaZulu-Natal, Private Bag X01, Scottsville, Pietermaritzburg 3209, South Africa; sibiyaj@ukzn.ac.za; 3 Agricultural Research Council, Vegetable, Industrial and Medicinal Plant, Private Bag X293, Pretoria 0001, South Africa; 4 Ethiopian Institute of Agricultural Research, Holeta Agricultural Research Centre, Addis Ababa P.O. Box 2003, Ethiopia; gemechukeneni@yahoo.com

**Keywords:** agro-morphological traits, cluster analysis, common bean, genetic diversity, principal component analysis

## Abstract

The objectives of this study were to assess the agronomic performance of common bean genotypes, previously selected for their response to infestation, by Mexican bean weevil and to identify promising lines that can be used as parents in a downstream breeding program. Field experiments were conducted using 144 genotypes under three different agro-ecologies in an unbalanced incomplete block design with three replications. Data on 15 agro-morphological traits were collected, and multivariate methods were used to examine the patterns of variation among the genotypes. The genotypes revealed a high level of phenotypic diversity for all agronomic traits. Six principal components, which contributed 84% of the total variation among the genotypes, were identified. The 15 agro-morphological traits classified the genotypes into three distinct major clusters and sub-clusters. The clustering patterns of the genotypes were according to the seed size, whereby the small and medium beans were distinctly separated from the large-seeded beans. The study established the existence of considerable genetic variations among common bean genotypes. Unique genotypes, such as Nasir, Awash Melka, and RAZ-36 from Cluster I, RAZ-2, RAZ-11, and RAZ-42 from Cluster II, and SER-125, SCR-15, MAZ-200, MAZ-203, and RAZ-120 from Cluster III, were selected based on their distinct agronomic performance. The selected genotypes could be useful for the common bean breeding program.

## 1. Introduction

The common bean (*Phaseolus vulgaris* L.) is the third most important source of calories, after maize and cassava, and the second most important source of dietary protein and minerals in the human diet [1]. In Africa, the major common bean-producing countries include Burundi, DR Congo, Ethiopia, Kenya, Rwanda, Tanzania, and Uganda, indicating that East Africa is the most suitable bean production region on the continent [1,2,3,4]. In Ethiopia, most of the traditional foods, especially during the fasting seasons, are prepared from pulse crops, such as chickpeas, field pea, faba beans, and lentils. However, recently there has been a growing interest in common beans, particularly among low-income farmers, since the prices of other highland pulses are rising [5,6].

The major common bean production areas are Oromiya, the Southern Nations, Nationalities, and Peoples (SNNP), and the Amahara regions. These regions cover about 98% (51% Oromiya, 27% SNNPR, and 20% Amhara) of the common bean production in the country [7]. Although farmers from different parts of the country grow different types of beans, the most predominant types being white and red small beans [3]. Both white and red small beans are produced in the Oromiya region and account for 61% and 44% of bean production in the country, respectively. In the Oromiya region, only two zones (East Shewa and West Arsi) cover 76% of the total bean production of the region [7]. In East Shewa, white beans (34%) are the most dominant types and are mainly grown for export, while in West Arsi, farmers only produce red beans [7,8].

The genetic improvement strategy of the National Common Bean Research Program in Ethiopia is focused mainly on consumer preferences and resistance to biotic and abiotic stresses. More than 55 improved common bean varieties have been released and adopted by farmers [9]. Despite the success of developing acceptable common bean genotypes, harnessing the genetic potential of the crop by delivering varieties with high yield and related quality traits is still hindered by the narrow genetic base used in the breeding program [10]. The National Bean Breeding Program relies mostly on exotic germplasms sourced from the Center for Tropical Agriculture (CIAT) and national breeding programs in neigbhoring countries.

Since the common bean’s introduction in the 16th century, farmers have been preserving and discovering important genotypes that are adapted to their local environments and needs, which has led to the evolution of morphologically diverse landraces [11,12]. Landraces have been used as a source of desirable genes in breeding for biotic and abiotic stresses [13]. A number of researchers have reported on the wide genetic diversity in the Ethiopian common bean genotypes for a number of important traits [3,14,15]. However, the potential of the local landraces as sources of breeding material is not yet well-known and exploited. The objective of the present study, therefore, was to assess the performance of common bean genotypes for yield and yield components across different agro-ecologies and to select promising parents for breeding.

## 2. Results

### 2.1. Agronomic Performance

The analyses of variance for each location revealed a highly significant variability (*p* < 0.001) among the genotypes for all the traits studied. In addition, the performance of the genotypes was highly influenced by the prevailing environment. Thus, a combined analysis of variance was conducted over three locations, which showed highly significant genotypes by environment interactions for all the traits (Table 1). The mean squares partitioned for genotype, environment, and genotype by environment interaction indicated that environment (location) effects were more important for the variability recorded in all the traits except for the pod per plant and hundred seed weight. The percentage contribution of the genotypic effect ranged from 0.9% for days to 50% flowering to 68% for pod per plant, while the environmental effects ranged from 99% to 13% for the above-mentioned traits. For pod per plant, the genotype main effect (68%) and genotype by environment interaction (19%) had more influence than the environment main effect. However, for the grain yield per plant, both the genotype (38%) and the environmental effects (42%) were important for the expression of the traits (Table 1). The coefficient of determination (R^2^) estimated for all the traits ranged from 0.82 for plant height to 0.99 for hundred seed weight.

The minimum, maximum, mean, and standard deviation, as well as the coefficient of variation values of the 15 agro-morphological traits recorded at the three locations, are presented in Table 2. The range for DTF was recorded from 37 to 52 days, with a mean of 44 days. The PH, LA, and TCC ranged from 32–56 cm, 0.80–5.80 m^2^, and 35–57 µmol/m^2^, respectively. The difference in DTM of late and early maturing genotypes was 25 days, with a mean value of 94 days, while GFP ranged from 37 to 59 days. The number of PPP and SPP ranged from 13–51 and 3–6, respectively. The genotypes revealed a high variation in seed size, ranging from small (12 g) to large (59 g). The minimum AGBM was 28 gm, and the maximum was 53 gm per plant, with the mean being 37 gm per plant. The GY showed a wide variation, with the values ranging from 15 to 42 gm per plant, and the mean yield was 26 gm per plant. In addition, the range of HI, BPR, and EGR were 40–92, 30–56, and 29–82, respectively. The coefficient of variation recorded in the traits studied ranged from 2.3% to 12.9%.

### 2.2. Principal Component Analysis (PCA)

The PCA grouped the 15 phenotypic traits into 15 components, which accounted for the entire (100%) variability among the studied genotypes. However, the principal components, with an eigenvalue of less than one, were eliminated. The first six principal components (PCs), accounting for 83.7% of the variability observed among the studied common bean genotypes, were maintained. Table 3 presents the eigenvectors and values, the percentage of total variance, and the total cumulative variance for the 15 phenotypic traits used in this study. The first principal component (PC1) explained 29.7% of the total phenotypic variation among the 144 common bean genotypes was mainly due to the additive effects of the GY, GPE, EGR, AGBM, and BPR. The second PC, which accounted for 19.4% of the total variation, was well associated with phenological traits, such as DTF, DTM, and the GFP. Likewise, the third PC, which accounted for about 11% of the total variance of the genotypes, was due to the discriminatory effect of the HSW and PPP. The variation in PH, DTF, and SPP constituted a large part of the total variation explained by the fourth PC. The fifth and sixth PCs accounted for 7.7% and 6.7% of the total variation, chiefly due to the contrast between the TCC and SPP, and HI and LA, respectively.

To select genotypes with the best performance, the contribution of each trait was determined by the PCA. It was found that yield had a significant effect on the phenotypic variation among the 144 genotypes. Hence, the top ten best genotypes were selected from both small and medium market classes based on grain yield performance. The mean performance of the top ten high-yielding genotypes from both small and medium-seeded genotypes is presented in Table 4.

Genotypes, such as Nasir, SER-125, Awash Melka, RAZ-36, 241757, 230526, RAZ-44, 241734, 214665, and NC-51, were selected from the small-seed market class, and they had a grain yield ranging from 29.2 to 41.8 g/plant. The top ten selected high-yielding genotypes from the medium market class included 207935, SCR-11, RAZ-40, NC-28, 211302, SCR-15, SCR-26, 228077, KK25/NAGAGA/19, and RAZ-120. These genotypes produced a grain yield ranging from 27.8 to 41.2 g/plant. There was no single genotype that showed consistent superiority for all the traits among the selected genotypes. However, the improved small-seeded variety, Nasir, exhibited the highest GY and GPE while genotype 207935 showed the highest AGBM, BPR, and EGR of all the tested genotypes. Based on the field performance of the 144 genotypes, 45% of the selected genotypes were landraces (241757, 230526, 241734, 214665, 207935, 211302, NC-51, NC-28, and 228077), 25% were resistant lines (RAZ-36, RAZ-44, RAZ-40, KK25/NAGAGA/19, and RAZ-120), 25% were released varieties (Nasir, SER-125, SCR-15, SCR-26, and Awash Melka) and 5% were advanced breeding lines (SCR-11).

### 2.3. Correlations of Yield and Its Components

The correlation among the 15 agro-morphological traits is presented in Table 5. Grain yield was highly significantly and positively (*p* < 0.001) correlated with AGBM, HI, GPE, BPR, and EGR. Similarly, GFP and PPP were highly significant (*p* < 0.01), and SPP and HSW had a significant (*p* < 0.05) correlation with GY. Biomass production rate was found to be negatively and highly significantly (*p* < 0.001) correlated with DTF, DTM, and GFP but highly (*p* < 0.001) positively correlated with AGBM and GPE. The total chlorophyll content, on the other hand, revealed a negative and significant association with DTF, DTM, GFP, and SPP. Similarly, HSW had a negative and significant correlation with LA, DTF, PPP, and SPP and a positive and significant association with PH. The days to 50% flowering had a significant negative association with HSW, GPE, and BPR. The relationship between PPP and SPP with HSW was also significant but negative.

### 2.4. Cluster Analysis

**Figure 1:**
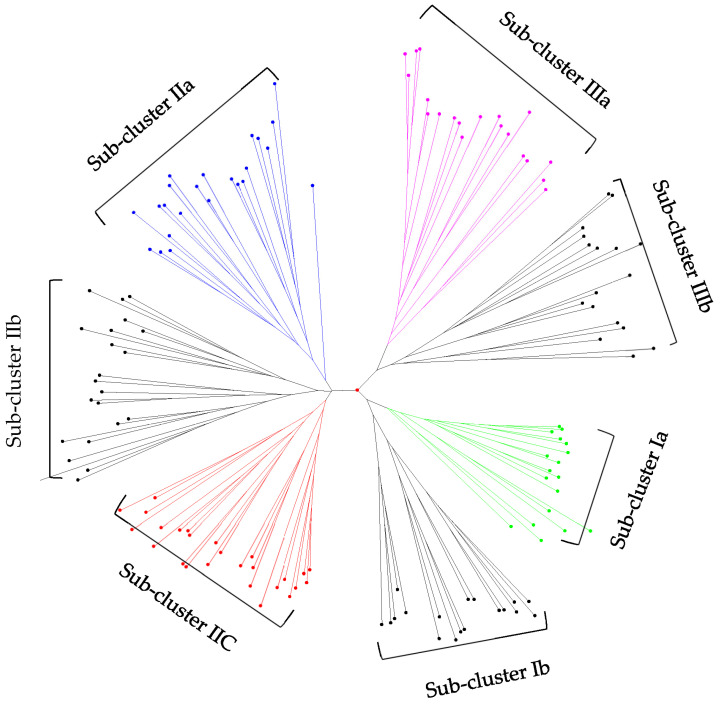
Dendrogram generated, based on hierarchical cluster analysis using UPGMA cluster algorithm, based on morphological data of 144 common bean genotypes (Supplementary Table S1).

The relationship among the 144 common bean genotypes was revealed by using the neighbor-joining algorithm using the unweighted pair group method (UPGMA). The cluster analysis on the mean of 15 phenotypic traits clearly classified the 144 genotypes into three major clusters and seven sub-clusters (Figure 1). The first cluster (Cluster I) was composed of 36 (25%) of the genotypes and was dominated by small-seeded beans. This cluster was further divided into two sub-clusters (sub-Cluster Ia and Ib), with 18 genotypes each. With regard to genotype status, Cluster I consisted of 26 landraces, two resistant lines, and five varieties. The second cluster (Cluster II) consisted of the largest number, mainly small-seeded genotypes (49%). This cluster was further sub-divided into three sub-clusters, with 26, 22, and 23 genotypes, respectively. Cluster III consisted mainly of large and medium-seeded genotypes. This cluster was comprised of 37 genotypes, which were further sub-divided into two sub-Clusters, with 20 and 17 genotypes, respectively. Of the 16 resistant lines, 50% were in Cluster III, together with large-seeded released varieties.

### 2.5. Performances of Genotypes in Different Clusters

Table 6 summarizes the cluster means of the 15 phenotypic traits for the three main clusters and seven sub-clusters. The mean performance of the clusters showed the presence of considerable phenotypic variation among genotypes within each cluster. Genotypes in Cluster I revealed the highest mean values for all the traits except for PH, HSW, and TCC. Genotypes in Cluster III had the highest mean values for PH, HSW, and TCC.

Sub-cluster Ia contained genotypes that had a large LA and a large number of SPP. Genotypes grouped in sub-Cluster Ib were characterized by tall plants with a large number of PPP, as well as the highest AGBM, GY, and EGR. Although sub-Clusters Ia and Ib consisted of genotypes with small-seed sizes, genotypes in sub-Cluster Ib were much smaller than those in sub-Cluster Ia. Genotypes in sub-Clusters IIa and IIb were relatively early maturing, with a short GFP. However, sub-Cluster IIc consisted of genotypes that were late maturing and took long to fully fill the grain. In general, the genotypes clustered in sub-Clusters IIa and IIc were low-performing genotypes that had an extended period of vegetative growth and the highest total chlorophyll content.

Out of the two sub-Clusters under Cluster III, sub-Cluster IIIb included the best-performing genotypes in traits, such as GY, HI, GPE, BPR, and EGR. These genotypes also had a high TCC, a short flowering time, and were of medium seed size. Sub-Cluster IIIa, on the other hand, consisted of tall genotypes with large seed sizes. The genetic distance averaged for all the genotypes in each cluster revealed that the genotypes in each respective cluster were diverse. The smallest mean genetic distance was observed among genotypes clustered in Cluster I sub-Cluster Ib, while the highest genetic distance was found among genotypes grouped in Cluster III sub-Cluster IIIa. Generally, cluster analysis allows the selection of unique and genetically complementary genotypes for breeding and conservation. Genotypes Nasir, Awash Melka, and RAZ-36 from Cluster I, RAZ-2, RAZ-11, and RAZ-42 from Cluster II, and SER125, SCR-15, MAZ-200, MAZ-203, and RAZ-120 from Cluster III were selected as potential parental genotypes. The selected genotypes have unique attributes, including grain yield, earliness, and seed color, shape, and size.

## 3. Discussion

### 3.1. Agronomic Performance

The present study examined the genetic variability and agronomic performance of 144 selected common bean genotypes for 15 yield and yield-related traits in three locations. The highly significant genotype mean squares for all the characters demonstrated that the genotypes exhibited a wide genetic variability for yield and yield-related traits. The observed highly significant environmental main effects suggested that the three locations were diverse in terms of weather- and location-related factors, such as temperature, rainfall, relative humidity, wind, altitude, soil physical and chemical properties. The three test locations represented three different agro-ecologies, with Melkassa representing the dryland agro-ecology, Arsi Negele representing the highly productive highland agro-ecology and Alem Tena representing the middling agro-ecology. Ceccarelli et al. [16] indicated that the genotype and environment components are recognized as the primary sources of variability in agronomic and genetic studies. Similarly, the highly significant genotype by environmental interaction indicated that genotypic performance is highly variable across different environments. Ceccarelli [17] also indicated that the expression of morphological and physiological plant characteristics associated with yield in optimal and stress conditions is different. Therefore, the discrimination and characterization of genotype adaptation across environments are crucial for optimizing the deployment of genetic resources.

In this study, the means and ranges of phenological traits and yield-related traits, such as the number of PPP, the number of SPP, HSW, and SW, revealed a wide range of genetic variation. A high phenotypic variation for these traits in the common bean was also reported by different authors [10,18,19,20,21,22,23,24]. The high phenotypic variation observed in this study may be attributed to the genetic variations among the genotypes and the environmental variations in the tested locations. In this study, more than 75% of the genotypes were landraces, suggesting that there was ample genetic variability among the landraces that can be exploited in future common bean improvement programs. This was also confirmed by other researchers that the Ethiopian common bean landraces were represented by high phenotypic diversity [3,10,24]. Similarly, the common bean grown in different parts of the world revealed a significant variation in yield and yield-related traits [18,20,25,26,27,28,29].

Principal component analysis (PCA)A Principal Component Analysis (PCA) was conducted to measure the relative contribution of each trait with regard to the total variation in the studied common bean genotypes. The first six components, with an eigenvalue of ≥1 explained 84% of the total variation were identified. However, about 50% of the phenotypic variation was explained by the first two components. Similar results were reported for agro-morphological traits in the common bean by several researchers [10,21,27,30,31]. In the present study, about 30% of the phenotypic variations observed were due to the variation in GY and AGBM. However, phenological traits also contributed significantly to discriminating the genotypes. The significant discriminatory effect of DTF was also reported by Burle et al. [21] and Fisseha [10]. Likewise, about 11% of the variations detected among the tested genotypes were due to the variation in seed weight. In previous studies, this trait was reported as the most important trait used to differentiate the two common bean gene pools [32]. However, the contribution of the trait in this study was relatively low compared to other previously reported results [10,21]. This could be due to the fact that most of the genotypes were selected from small (74%) and medium (15%) seed sizes, as reported by De Lima et al. [27].

The top 20 common bean genotypes were selected as potential parents for breeding programs, based on PCA1 values, which constitute the additive effect of GY, GPE EGR, and AGBM. The principal component analysis showed that grain yield had the most significant role in discriminating the 144 genotypes. The selection of the top genotypes was conducted according to the common bean market preferences in the major common bean-producing regions in Ethiopia, where the Mesoamerican beans (small-seeded) have more market demand than the Andean (large-seeded) genotypes. Based on their agronomic performance, the selected genotypes were composed of nine landraces, five resistant lines, three varieties, and three advanced breeding lines. As can be expected, the released varieties in the selected small-seeded group topped the rank in grain yield. The majority (45%) of the selected genotypes were landraces, suggesting that landraces can be used as a good source of valuable genes for future common bean breeding programs in Ethiopia [33]. Although the local landraces were found to be better adapted, genetically diverse, and agronomically suitable, the National Bean Breeding Program has been entirely dependent on the exotic germplasm. The SCR lines (SCR-11 and SCR-15) were the two top selected genotypes from the medium-sized red bean group. These lines are red beans that were developed for drought-prone areas carrying drought tolerance and with recessive genes for resistance to bean common mosaic virus [34]. The lines with Zabrotes-resistance genes, such as RAZ-36, RAZ-40, RAZ-44, and RAZ-120, and the Malawian resistance variety (KK25/MAIAWA/19), were found to be agronomically suitable.

### 3.2. Correlations of Yield and Its Components

Yield is a complex trait and is the outcome of the interaction of a number of genes and traits. Moreover, the expression of the traits is highly influenced by environmental factors, such as temperature, moisture, and light. It is also well known that the overall yield performance of genotypes is determined by the interaction of the traits rather than the expression of individual traits [16]. Blum [35] also indicated that yield per se is not under direct genetic control but under the control of the integrated effects of a multitude of physiological and biochemical processes. Hence, an understanding of the association between yield and yield-related traits is very crucial in order to exploit the genetic variability through selection. In the present study, grain yield had a significant positive association with the GFP, the number of PPP, HSW, AGBM, and HI. A selection based on these traits can be used as an indirect selection criterion for the improvement of grain yield in the common bean. Several researchers have also reported the positive significant correlation of grain yield with the above-mentioned traits [10,24,25,28,36,37]. Different authors [10,34,37,38] have also reported a strong positive correlation between HSW and GY. Some reports, on the other hand, have indicated a strong negative correlation between GY and HSW [24,28,39,40]. The variation in the sets of traits and the strength of the association might be a result of the variations in the environmental conditions and the genotypes used.

### 3.3. Cluster Analysis

The hierarchical cluster analysis conducted on the means of 15 agro-morphological traits resulted in three distinct major clusters and seven sub-clusters. For the traits under consideration, the within-cluster variation was found to be the lowest, while the between-cluster variation was the highest [41,42]. The mean performance of the genotypes grouped under the different clusters and sub-clusters showed considerable phenotypic variation. The clustering patterns were according to the seed size, where small and medium-seeded genotypes were clustered in Cluster I and II, while all the large-seeded genotypes were grouped in Cluster III. Several authors, such as Singh et al. [43], Burle et al. [21], Madakbaş and Ergin [44], and Boros et al. [20], support the present result. Based on hundred-seed weight, genotypes with HSW < 25 g are categorized as small-seeded, HSW ≥ 25–41 g as medium- seeded and HSW > 41 g as large-seeded. The clustering of genotypes, based on their seed size (gene pools), was clearly observed in the molecular genetic diversity analysis using SNP markers [45]. The clustering of landraces across all clusters indicated that Ethiopian landrace collections had a wide genetic variation for yield and yield-related traits. In addition, a large number (82%) of the genotypes was found to have a small to medium seed size, suggesting that the Ethiopian common bean genotypes are predominantly from the Mesoamerican gene pool, as supported by Asfaw et al. [3].

## 4. Materials and Methods

### 4.1. Description of the Study Site

**Figure 2:**
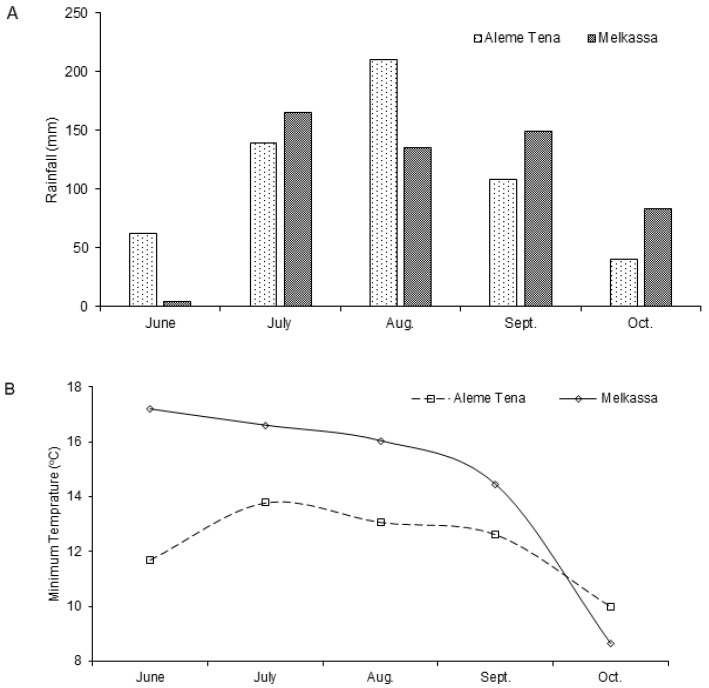

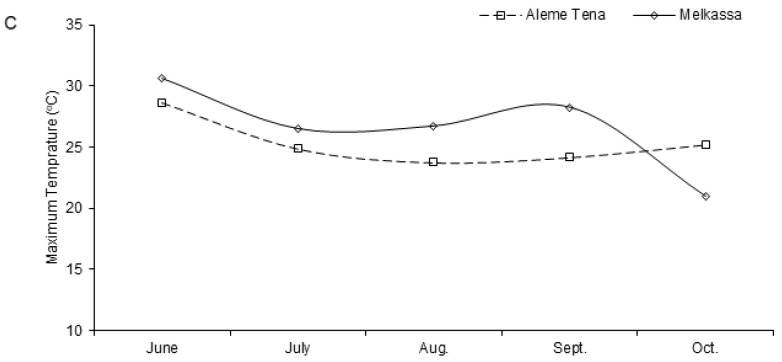
Climate data (**A**) rainfall (in mm), (**B**) minimum and (**C**) maximum temperatures (in °C) of Melkassa and Alem Tena sites during the growing.

The study was conducted at three on-station trial sites in the Oromiya region of central Ethiopia. The sites were Melkassa (8°24′52.04″ N, 39°19′41.22″ E), Alem Tena (8°17′32.29″ N, 38°56′48.77″ E), and Arsi Negele (7°22′30.29″ N, 38°40′17.78″ E), which are located at an altitude of 1550, 1611, and 1960 meters above sea level (m.a.s.l.), respectively. The climatic data of Melkassa and Alem Tena were collected from Melkassa and Debrie Zeit Agricultural Research Centers, respectively. However, the weather station at Arsi Negele was not functional, and the weather data is not included in this study. The weather data on rainfall and temperature for the two sites are presented in Figure 2. The soil types of Melkassa and Alem Tena are sandy loamy, while the soil is clay in Arsi Negele.

### 4.2. Experimental Material and Experimental Design

A total of 144 common bean genotypes were selected on the basis of the prior screening of the genotypes for their response to bruchid infestation under laboratory conditions. The genotypes comprised 109 landraces, 16 released varieties, and 19 pre-release breeding lines. The 109 common bean landraces were collected from different regions of Ethiopia, and of the 19 pre-released genotypes, 16 were resistant to the Mexican bean weevil. The genotypes were grown during the off-season under irrigation for seed increase and to offset any differences in seed age and the effects of the prior growing environments [46]. The 144 genotypes were planted in a 12 × 12 alpha lattice design with three replications. The common bean genotypes were planted in 3 m long three rows, an inter-row spacing of 1 m, and an intra-row spacing of 40 cm. Weeds were controlled by frequent hand-weeding throughout the experimental period. Di-ammonium phosphate (DAP) fertilizer was applied during planting at a rate of 100 kg/ha [47], and other agronomic practices were carried out according to the cultivation practices recommended for each site.

### 4.3. Data Collection

In this study, a total of 15 phenological and agronomic traits were evaluated based on the IBPGR [48] common bean descriptors. For the agronomic traits, five randomly selected plants were sampled for data collection, while the phenological traits, such as days to 50% flowering (DTF) and days to 90% maturity (DTM), were recorded on a whole plot basis. Data on the following agronomic traits were collected: Plant height (PH), pods per plant (PPP), seeds per pod (SPP), hundred seed weight (HSW), the aboveground biomass (AGBM), and grain yield (GY).

In addition, the harvest index (HI) was measured as a proportion of grain yield to the aboveground biomass, and the grain-filling period (GFP) was calculated by subtracting the number of days to 90% maturity from the days to 50% flowering. Grain production efficiency (GPE) was calculated as a proportion of the grain-filling period to the duration of the vegetative period, and biomass production rate (BPR) was estimated by dividing the aboveground biomass weight by the days to 90% physiological maturity. Economic growth rate (EGR) was calculated as a proportion of grain yield to the grain seed fill period. Other physiological parameters, such as leaf area (LA) measured by a leaf area meter (LICOR model LI-3000) and total chlorophyll content (TCC) measured by a non-destructive, hand-held chlorophyll meter (SPAD-502 Chlorophyll Meter), were also included.

### 4.4. Data Analysis

Data were subjected to the analysis of the unbalanced incomplete block design procedure using GenStat Version 19 [49]. The homogeneity of variances among the three locations was examined by using Bartlett’s test for each of the studied agro-morphological traits. Bartlett’s test showed that all the traits had an equal error variance. All the agro-morphological traits were checked successively for normality using GenStat, and all the traits showed a normal distribution. The three locations were treated as environments, and a combined analysis of variance over the environments was done to estimate the variance component. Genotypes and environments were considered as fixed effects and replications, and blocks as random effects, and a combined analysis over environments was estimated from the linear additive model, which is expressed as:

$$Y_{ijklm}=\mu +r_{i}+b_{j}+\varphi_{k}+G_{l}+E_{m}+{GE}_{lm}+\varepsilon_{ijklm}$$

where *μ* = the overall mean, *r_i_* = the effect due to *i*^th^ replication, *b_j_* = the effect due to the *j*^th^ block within the *i*^th^ replication, *φ_k_* = the effect due to the *k*^th^ incomplete block within the *j*^th^ block, *G_l_* = genotypic effect of the *l*^th^ genotype, *E_m_* = environmental effect of the *m*^th^ environment, *GE_lm_* = the interaction effect of the *l*^th^ genotype and the *m*^th^ environment.

The data were also subjected to the Principal Component Analysis (PCA) procedure using Genstat Version 19. For multivariate analysis, the data were standardized to a mean of zero, and a variance of unity was made to avoid the differences in scales used for recording data on the different characters [50]. The top ten highest-yielding genotypes were selected based on the traits that had the highest contribution to the first principal component, i.e., grain yield.

The correlation coefficients between characters were estimated based on the following formula:

r=Covxysqrtσx2+σy2

where Cov_xy_ = co-variance of traits x and y, σ_x_^2^ = variance of x and σ_y_^2^ = variance of y.

A hierarchical cluster analysis was performed to examine the grouping patterns of the genotypes based on their dissimilarity matrix with respect to the corresponding means of all the fifteen characters. The dissimilarity matrix was calculated using the Dice similarity index [51], and the cluster analysis was done by using the unweighted pair group method, the arithmetic mean (UPGMA), using DARwin 6.0 software [52]. A dendrogram was then generated on the dissimilarity matrix, and a bootstrap analysis was performed for node construction using 10,000 bootstrap values. The group means for all 15 agro-morphological traits were calculated and compared. Promising parental genotypes were selected.

## 5. Conclusions

The study identified a considerably wide genetic diversity among the 144 common bean genotypes for all the 15 phenotypic traits studied. Traits such as the GY, HSW, and AGBM were found to be the most important traits in differentiating germplasm into different clusters. It was also found that the Ethiopian common bean landraces showed a wide range of variation for all 15 of the agro-morphological traits studied, which suggests these germplasms can be used as valuable sources of genes in the National Common Bean Improvement programs. Genetically unique genotypes, such as Nasir, Awash Melka, and RAZ-36 from cluster I RAZ-2, RAZ-11, and RAZ-42 from Cluster II and SER-125, SCR-15, MAZ-200, MAZ-203, and RAZ-120 from Cluster III, were identified as suitable parental genotypes. Released varieties, Nasir and Awash Melka, are the top high-yielding varieties that have been adopted in most of the bean growing areas. SER-125 and SCR-15, on the other hand, is a recently released variety that possesses most of the farmers’ preferred traits. The selected genotypes could be useful for the common bean-breeding program.

## Figures and Tables

**Table 1:** Combined analysis of variance of 15 agro-morphological traits recorded on 144 common bean genotypes at three locations.

Traits	Mean Square							
	Replication (DF = 2)	Block (DF = 11)	iBlock (DF = 11)	Genotype (G) (DF = 121)	Environment (E) (DF = 2)	G × E Interaction (GEI) (DF =286)	Error (DF = 862)	R^2^
Days to 50% flowering (DTF)	1.87	71.38	44.09	49.00 **	5174.08 **	8.99 **	2.41	0.93
Plant height (PH)	219.97	153.7	116.38	204.82 **	19,842.36 **	197.00 **	32.57	0.82
Total chlorophyll content (TCC)	227.23	386.6	106.05	111.18 **	2050.41 **	65.64 **	4.78	0.91
Leaf area (LA)	0.59	2.02	1.47	1.50 **	224.60 **	1.02 **	0.04	0.97
Days to 90% maturity (DTM)	50.43	539.4	124.7	182.58 **	19,915.01 **	54.92 **	4.55	0.96
Grain filling period (GFP)	33.71	351.6	89.16	137.80 **	6043.10 **	51.46 **	6.41	0.9
Pods per plant (PPP)	55.55	971.8	682.06	540.76 **	105.54 **	148.81 **	7.41	0.95
Seeds per pod (SPP)	0.77	14.49	8.91	4.24 **	9.17 **	1.80 **	0.21	0.88
Hundred seed weight (HSW)	12.83	2571	536.61	675.19 **	589.14 **	24.16 **	2.1	0.99
Aboveground biomass (AGBM)	30.2	300.9	88.8	153.36 **	4539.12 **	82.36 **	4.14	0.94
Grain yield (g/plants) (GY)	24.28	568.1	281.35	219.75 **	240.55 **	111.65 **	4.3	0.95
Harvest index (HI)	15.62	1620	1042.57	783.12 **	12,005.66 **	708.42 **	23.46	0.95
Grain production efficiency (GPE)	60.61	915.4	558.21	436.05 **	1359.47 **	184.89 **	10.54	0.93
Biomass production rate (%) (BPR)	17.38	652.6	75.51	164.86 **	1352.50 **	102.99 **	5.15	0.93
Economic growth rate (%) (EGR)	37.79	2672	832.04	851.69 **	2826.06 **	483.27 **	24.76	0.93

** Significant at *p* < 0.001.

**Table 2:** Summary statistics on 15 agro-morphological traits evaluated on 144 common bean genotypes at three locations.

Trait	Min	Max	Mean ± SE	SD	CV%
Days to 50% flowering (Days)	37	51.9	44.17 ± 0.12	1.55	3.51
Plant height (cm)	31.7	56.2	44.20 ± 0.30	5.71	12.91
Total chlorophyll content (µmol/m^2^)	35	57.1	45.01 ± 0.17	2.19	4.86
Leaf area (m^2^/plant)	0.8	5.8	3.00 ± 0.03	0.19	6.39
Days to 90% maturity (Days)	79.1	103.8	94.41 ± 0.23	2.13	2.26
Grain filling period (Days)	37.4	58.6	50.25 ± 0.18	2.53	5.04
Pods per plant (No)	13.1	50.9	27.70 ± 0.28	2.72	9.83
Seeds per pod (No)	2.1	6.1	4.21 ± 1.06	0.46	10.84
Hundred seed weight (gm)	12.1	58.8	24.84 ± 0.27	1.45	5.84
Above ground biomass (gm/plants)	27.7	53.2	36.74 ± 0.19	2.04	5.54
Grain yield (gm/plant)	14.6	41.8	25.64 ± 0.21	2.07	8.09
Harvest index	39.76	92.1	69.99 ± 0.47	4.84	6.92
Grain production efficiency (gm/plants)	14	54.5	29.52 ± 0.28	3.25	11
Biomass production rate (%)	30.3	56.2	39.18 ± 0.20	2.27	5.79
Economic growth rate (%)	28.5	82	51.48 ± 0.43	4.98	9.67

**Table 3:** Principal component (PC) analysis of various agro-morphological traits estimated at three locations.

Trait	PC1	PC2	PC3	PC4	PC5	PC6
AGBM	0.41	0.00	−0.03	0.17	−0.22	0.19
BPR	0.38	−0.23	0.06	0.15	−0.24	0.15
DTF	−0.12	0.31	0.02	0.51	−0.26	−0.30
DTM	0.02	0.55	−0.21	0.06	0.10	0.05
EGR	0.42	−0.14	0.13	0.11	−0.04	−0.16
GFP	0.10	0.46	−0.25	−0.23	0.26	0.23
GPE	0.43	0.12	−0.08	−0.18	0.19	0.08
GY	0.45	0.04	0.02	0.01	0.05	−0.06
HI	0.19	0.11	0.05	−0.27	0.22	−0.64
HSW	0.10	−0.21	−0.63	−0.02	0.04	0.05
LA	0.09	0.27	0.16	0.07	−0.22	0.44
PH	0.09	0.13	−0.38	0.50	−0.04	−0.27
PPP	0.10	0.26	0.52	0.21	0.31	0.01
TCC	0.09	−0.27	0.08	0.22	0.48	−0.02
SPP	0.11	0.16	0.08	−0.42	−0.53	−0.28
Eigenvalue	4.45	2.91	1.68	1.36	1.15	1.00
% total variation	29.67	19.37	11.23	9.09	7.65	6.66
% cumulative variation	29.67	49.04	60.27	69.36	77.01	83.67

AGBM = aboveground biomass (gm/plant); BPR = biomass production rate; DTF = days to 50% flowering (days); DTM = days to 90% maturity (days); EGR = economic growth rate (%); GFP = grain filling period (days); GPE = grain production efficiency (gm/plant); GY = grain yield (gm/plant); HI = harvest index; HSW = hundred seed weight (gm/100 seed); LA = leaf area (m^2^/plant); PH = plant height (cm); PPP = pods per plant (No); TCC = total chlorophyll content (µmol/m^2^); SPP = seeds per pod (No).

**Table 4:** Mean performance of the top ten selected common bean genotypes for seed color and yield and yield-related traits.

Genotype	SC	DTF	PH	TCC	LA	DTM	PPP	SPP	HSW	AGBM	GY	HI	GPE	BPR	EGR	GFP
**Top ten small-seeded genotypes**																
Nasir	Red	41.6	53.3	48.0	2.5	95.4	36.0	5.1	24.6	46.1	41.8	63.5	54.5	48.3	77.5	53.9
SER-125	Red	41.8	41.7	47.1	2.6	90.7	26.0	3.5	25.6	39.7	36.5	77.3	42.6	44.3	75.9	48.9
Awash Melka	White	46.6	52.8	49.1	3.0	93.9	33.7	4.5	21.7	46.6	34.6	63.5	34.0	52.6	76.9	47.3
RAZ-36	White	42.7	45.0	53.4	3.2	96.2	46.3	3.1	18.1	45.5	33.1	66.9	41.3	47.1	63.3	53.6
241757	Red	47.0	47.2	41.5	2.9	95.7	29.8	4.4	22.7	43.7	32.9	76.3	34.0	45.6	68.7	48.7
230526	Red	42.9	41.1	40.4	3.4	96.6	27.0	5.0	23.6	37.0	32.2	86.9	40.3	38.3	59.8	53.7
RAZ-44	White	42.8	48.3	50.1	2.9	96.2	31.2	4.1	18.1	42.1	31.4	82.5	39.2	43.8	60.7	53.4
241734	Red	43.4	46.1	45.6	4.0	101.1	30.0	4.6	22.1	44.4	31.3	72.1	41.6	44.0	54.5	57.7
214665	Red	43.1	44.4	43.7	3.4	99.3	27.4	5.4	22.8	41.1	30.1	74.6	39.5	41.4	53.7	56.2
NC-51	Red	42.1	41.1	42.9	2.6	95.1	26.6	3.8	24.1	38.2	29.2	74.9	37.1	40.0	54.9	53.0
**Top ten medium-seeded genotypes**																
207935	Carioca	44.9	51.1	49.6	3.2	95.6	24.2	5.7	29.4	53.2	41.2	80.7	46.8	56.2	82.0	50.7
SCR-11	Red	42.0	45.0	49.9	2.7	92.3	25.4	3.9	29.2	44.9	36.9	56.6	44.2	48.8	74.3	50.3
RAZ-40	White	41.4	37.8	49.5	3.1	89.6	20.3	3.7	36.7	35.8	32.6	62.4	32.2	40.6	60.7	48.1
NC-28	Cream	40.9	45.0	47.3	3.1	99.4	32.0	3.1	28.9	42.3	31.8	75.8	45.0	42.6	55.0	58.6
211302	Brown	39.8	38.3	47.8	2.8	89.0	21.6	4.2	36.5	42.3	31.7	77.7	39.0	47.2	66.1	49.2
SCR-15	Red	43.3	38.9	47.6	2.8	94.0	27.1	3.7	38.3	41.5	31.3	89.0	36.5	43.8	62.1	50.7
SCR-26	Red	43.6	49.4	47.2	3.0	92.6	23.9	4.2	27.7	42.9	29.2	67.5	31.8	46.1	57.8	49.0
228077	Red	42.9	43.3	37.5	3.4	100.7	26.3	5.7	25.9	38.4	28.4	75.8	39.3	38.1	48.8	57.8
KK25/MAIAWA/19	Red	43.6	47.2	42.0	2.8	95.4	20.8	5.6	36.9	33.1	28.2	77.3	33.7	34.9	54.7	51.9
RAZ-120	White	45.7	45.0	50.3	2.8	90.7	28.6	3.7	26.4	38.3	27.8	75.1	27.4	42.6	63.1	45.0

SC = seed color; DTF = days to 50% flowering (days); PH = plant height (cm); TCC = total chlorophyll content (µmol/m^2^); LA = leaf area (m^2^/plant); DTM = days to 90% maturity (days); PPP = pods per plant (No); SPP = seeds per pod (No); HSW = hundred seed weight (gm/100 seed); AGBM = aboveground biomass (gm/plant); GY = grain yield (gm/plant); HI = harvest index; GPE = grain production efficiency (gm/plant); BPR = biomass production rate (%); EGR = economic growth rate (%), GFP = grain filling period (days).

**Table 5:** Correlation analysis among 15 agro-morphological traits in 144 common bean genotypes recorded at three locations.

Trait	DTF	PH	TCC	LA	DTM	GFP	PPP	SPP	HSW	AGBM	HI	GPE	BPR	EGR	GY
DTF	1.00														
PH	0.31 ***	1.00													
TCC	−0.26 **	0.05	1.00												
LA	0.14	0.03	−0.12	1.00											
DTM	0.52 ***	0.29 ***	−0.33 ***	0.31 ***	1.00										
GFP	0.03	0.16	−0.23 **	0.28 ***	0.87 ***	1.00									
PPP	0.21 *	−0.04	0.04	0.24 **	0.27 ***	0.20 *	1.00								
SPP	−0.02	−0.04	−0.24 **	0.14	0.14	0.17	−0.04	1.00							
HSW	−0.24 **	0.25 **	0.11	−0.22 **	−0.10	0.01	−0.61 ***	−0.18 *	1.00						
AGBM	−0.09	0.22 **	0.15	0.24 **	0.09	0.15	0.10	0.22 **	0.18 *	1.00					
HI	−0.07	0.06	0.08	0.07	0.12	0.18 *	0.13	0.26 **	−0.01	0.14	1.00				
GPE	−0.34 ***	0.12	0.05	0.15	0.26 **	0.50 ***	0.23 **	0.21 *	0.20 *	0.69 ***	0.40 ***	1.00			
BPR	−0.30 ***	0.11	0.27 **	0.09	−0.34 ***	−0.22 **	−0.01	0.12	0.19 *	0.88 ***	0.08	0.55 ***	1.00		
EGR	−0.16	0.09	0.19 *	0.01	−0.22 **	−0.16	0.21 *	0.12	0.15	0.70 ***	0.31 ***	0.72 ***	0.77 ***	1.00	
GY	−0.16	0.15	0.09	0.12	0.12	0.23 **	0.26 **	0.18 *	0.18 *	0.75 ***	0.39 ***	0.92 ***	0.68 ***	0.92 ***	1.00

PH = plant height (cm); LA = leaf area (m^2^/plant); TCC = total chlorophyll content (µmol/m^2^); DTF = days to 50% flowering (days); DTM = days to 90% maturity (days); PPP = pods per plant (No); SPP = seeds per pod (No); HSW = hundred seed weight (gm/100 seed); AGBM = aboveground biomass (gm/plant); GY = grain yield (gm/plant); HI = harvest index; GPE = grain production efficiency (gm/plant); BPR = biomass production rate (%); EGR = economic growth rate (%), GFP = grain filling period (days). *** = significant (*p* < 0.001); ** = significant (*p* < 0.01); * = significant (*p* < 0.05).

**Table 6:** The cluster means of 15 agro-morphological traits for the common bean genotypes, based on data recorded at three locations.

Trait	Cluster Means						
	C-I (*n* = 36)		C-II (*n* = 71)		C-III (*n* = 37)		
	SC-Ia (*n* = 18)	SC-Ib (*n* = 18)	SC-IIa (*n* = 26)	SC-IIb (*n* = 22)	SC-IIc (*n* = 23)	SC-IIIa (*n* = 20)	SC-IIIb (*n* = 17)
PH	43.4	47.2	43.0	40.8	44.2	47.1	44.9
LA	3.41	3.13	3.15	2.82	3.01	2.91	2.87
TCC	44.3	45.7	43.3	45.9	44.2	44.7	48.0
DTF	43.2	45.4	44.3	43.9	45.6	44.1	42.2
DTM	95.7	96.2	94.6	87.4	99.1	94.7	93.1
GFP	52.4	50.9	50.4	43.6	53.6	50.6	50.9
PPP	27.0	37.8	25.5	26.0	34.3	17.9	26.1
SPP	4.8	4.3	4.4	4.0	4.2	3.6	4.2
HSW	23.2	19.7	21.4	21.1	16.9	42.2	32.4
AGBM	38.8	41.3	36.2	33.6	32.8	36.1	40.8
GY	28.2	32.2	22.8	21.9	22.0	25.3	30.7
HI	75.8	75.6	64.6	65.9	68.7	67.2	76.6
GPE	34.5	36.3	26.1	21.8	25.9	29.5	37.1
BPR	40.5	43.2	38.7	38.7	33.3	38.2	44.0
EGR	53.9	64.2	45.6	50.8	41.6	49.6	61.0
Genetic distance	0.45	0.53	0.51	0.59	0.56	0.60	0.58

PH = plant height (cm); LA = leaf area (m^2^/plant); TCC = total chlorophyll content (µmol/m^2^); DTF = days to 50% flowering (days); DTM = days to 90% maturity (days); PPP = pods per plant (No); SPP = seeds per pod (No); HSW = hundred seed weight (gm/100 seed); AGBM = aboveground biomass (gm/plant); GY = grain yield (gm/plant); HI = harvest index; GPE = grain production efficiency (gm/plant); BPR = biomass production rate (%); EGR = economic growth rate (%), GFP = grain filling period (days); C = cluster; SC = sub cluster; *n* = number.

## Data Availability

Data is contained within the article.

## Supplementary Materials

The following supporting information can be downloaded at: https://www.mdpi.com/article/10.3390/plants12122342/s1, Table S1: Lists of genotype in each Cluster and Sub-cluster.
