# Supplementary material for: Agromorphological and Physiological Performance of Ethiopian Common Bean (Phaseolus vulgaris L.) Genotypes under Different Agroecological Conditions
Source: Plants (Basel). 2023 Jun 16;12(12):2342. doi: 10.3390/plants12122342 (PMC10305400; doi:10.3390/plants12122342)
Supplement: Supplementary file 1 [file plants-12-02342-s001.zip › plants-2383999-supplementary.pdf]

Supplementary Table S1. Lists of genotype in each Cluster and Sub-cluster.

**Cluster I**		**Cluster II**			**Cluster III**	
**Sub-Cluster Ia**	**Sub-Cluster Ib**	**Sub-Cluster IIa**	**Sub-Cluster IIb**	**Sub-Cluster IIc**	**Sub-Cluster IIIa**	**Sub-Cluster IIIb**
211284	207938	201066	208702	207534	208638	207935
211361	208703	207934	211267	208367	208699	208995
214663	211304	211266	211269	208705	211311	211302
214664	211314	211286	211279	211315	219231	237080
214665	211340	211305	211280	211320	223329	NC-13
228077	213197	211323	211347	211325	NC-14	NC-28
230526	214675	211346	211356	211331	NC-15	RAZ-120
237993	228812	211348	211362	211333	NC-18	RAZ-9
241734	241756	211349	211546	212860	NC-25	KK25/MAIAWA/19
241736	241757	215048	211552	214676	NC-29	Ayenew
241748	NC-30	215049	213046	215391	NC-34	Gofta
NC-05	NC-49	215051	214678	215720	NC-52	Kufanzik
NC-07	RAZ-36	228082	215719	228086	RAZ-40	Ser 125
NC-12	RAZ-44	228085	230525	228813	KK25/NAGAGA/184	Wedo
NC-17	Awash Melka	228522	NC-10	228911	MAZ 153	SCR-11
NC-51	Beshbesh	228913	NC-54	230044	MAZ 200	SCR-15
NC-53	Nasir	241739	NC-61	230661	MAZ 203	SCR-26
Red wolayta	Roba	241752	RAZ-11	232196	Deme	
		244805	RAZ-11-1	237079	DRK	
		NC-16	RAZ-2	241134	Tinkie	
		NC-20	SMARC 4	NC-44		
		NC-39	KAT-B1	NC-57		
		NC-50		Awash-1		
		RAZ-42				
		Mexican 142				
		RAZ-28-8				
